# Association between advance care planning and depressive symptoms among community-dwelling people with dementia: An observational cross-sectional study during the COVID-19 pandemic in Japan

**DOI:** 10.3389/fpubh.2023.915387

**Published:** 2023-03-30

**Authors:** Miharu Nakanishi, Taeko Nakashima, Yuki Miyamoto, Mai Sakai, Hatsumi Yoshii, Syudo Yamasaki, Atsushi Nishida

**Affiliations:** ^1^Department of Psychiatric Nursing, Tohoku University Graduate School of Medicine, Sendai-shi, Japan; ^2^Mental Health Promotion Unit, Research Center for Social Science and Medicine, Tokyo Metropolitan Institute of Medical Science, Setagaya-ku, Japan; ^3^Department of Social Healthcare and Business, Faculty of Healthcare Management, Nihon Fukushi University, Mihama-cho, Japan; ^4^Department of Psychiatric Nursing, School of Health and Nursing, Graduate School of Medicine, The University of Tokyo, Bunkyo-ku, Japan

**Keywords:** advance care planning, community, dementia, depression, family caregiver

## Abstract

**Objectives:**

Advance care planning (ACP) is an increasing priority for people with dementia during the COVID-19 pandemic. This study evaluated the association between ACP initiation and depressive symptoms among home-dwelling people living with dementia.

**Methods:**

An internet-based questionnaire survey was conducted with Japanese family caregivers of home-dwelling persons with dementia in June 2021. Family caregivers evaluated the level of depressive symptoms in persons with dementia using the Neuropsychiatric Inventory (NPI). Caregivers also rated the quality of life of persons with dementia using the EQ-5D-5L.

**Results:**

A total of 379 family caregivers participated in the survey. Depressive symptoms were reported in 143 persons with dementia (37.7%). A total of 155 persons with dementia (40.9%) had initiated ACP, of which 88 (56.8%) had care professionals involved in ACP conversation. After adjusting for the characteristics of persons with dementia and caregivers, persons with professional involvement showed significantly more severe depressive symptoms compared to those who did not initiate ACP. There was no significant difference in the quality of life of persons with dementia according to ACP initiation.

**Conclusions:**

Many home-dwelling persons with dementia experienced depressive symptoms during the COVID-19 pandemic, especially in cases where care professionals were involved in ACP conversations. Optimal and proactive ACP approaches need to be developed to prevent depressive symptoms in newly diagnosed persons.

## 1. Introduction

Living well with dementia becomes a global public health priority as the number of people with dementia continues to increase (1). Dementia is currently irreversible and people with dementia face a progressive decline in functional and mental capacity, with a median survival of 3–10 years following clinical diagnosis (2, 3). Although promotion of early diagnosis is often included in several national dementia plans (4), dementia diagnosis is associated with an elevated risk of depression and suicide (5–7). Suicidal ideation may be caused by dementia-related anxiety (8). Post-diagnostic support should be embedded in dementia plans to support people with dementia in re-establishing and maintaining a positive identity in the face of the condition (9).

Timely advance care planning (ACP) could comprise post-diagnostic support for people with dementia. ACP is defined as an ongoing communication process about future care among the person affected by dementia, family member/s, and the healthcare team (10). Initiation of ACP following dementia diagnosis provides an opportunity for people with dementia to express their values in life. Such communication and expression might prevent the development of depressive symptoms and reduce the risk of suicide. However, ACP for people with dementia is typically reported in care home settings (11, 12), for those with moderate to severe cognitive impairment who may lack decisional capacity (13). This results in their family members having to engage in decision making processes for them (13). The active involvement of community-dwelling people with dementia in ACP has not been sufficiently researched. Furthermore, outcomes of ACP are typically focused on end-of-life care measures (14). Little is known about the impact of ACP on depressive symptoms in persons with dementia.

ACP is an urgent priority for people with dementia during the COVID-19 pandemic. COVID-19 has caused a global health crisis alongside enforced isolation measures, which have a disproportional impact on people with dementia (15). Being unable to access social support services due to COVID-19 has contributed to worse quality of life in people with dementia (16). Furthermore, these individuals are particularly vulnerable to COVID-19 because of their age, multimorbidity, and difficulties in maintaining physical distancing (17). Thus, ACP is recommended to discuss the stage at which hospital admission for COVID-19 might not add much value (18). This may affect the practice of ACP among persons with dementia and their family caregivers. Moreover, understanding the association between depression and ACP during the COVID-19 pandemic can provide implications for imparting dementia care in adapted formats, considering the long-term public health restrictions.

This study therefore investigated the association between ACP initiation and depressive symptoms among people with dementia during the COVID-19 pandemic. We hypothesized that individuals having initiated ACP would show lower levels of depressive symptoms compared to those who have not yet initiated ACP.

## 2. Materials and methods

### 2.1. Design

This research was a cross-sectional observational study that was conducted using an online survey hosted by an Internet survey company (Macromill Inc.), which provides a global online research system. This web-based survey was conducted with Japanese residents. For more detailed information, please refer to Nakanishi et al. (19) study.

### 2.2. Setting

On June 25, 2021, a self-administered questionnaire was distributed to eligible individuals who were registered members of the survey company. Participants aged 40 years or older were randomly sampled from the company's member pool and were asked to complete the questionnaire by 27 June 2021. A continuous rise in the daily number of COVID-19 cases was observed during the study period, following the relief of the emergency declaration on 20 June. By 24 June, 21.3% of older adults in Japan had received the second dose of COVID-19 vaccine (20).

The members' continued participation was considered indicative of their consent to the questionnaire instructions provided on the website. The instructions assured the participants that their personal information would be protected and that their data would be anonymized. Any identifying information (participants' names and other identifiers that could lead to the identification of a participant) was removed when we received the data from the Internet survey company, and no images/videos were obtained from the participants.

### 2.3. Participants

Potential participants fulfilled the following criteria: (a) aged 40 years or older, (b) having been a primary non-professional caregiver for a home-dwelling person with dementia, and (c) having no conflicts of interest with advertising or marketing research entities. We excluded caregivers under the age of 40 because they comprise only 2% of all caregivers in Japan, making it difficult to consider such young caregivers providing care (21). Dementia in this survey was defined as having formal diagnosis of Alzheimer's disease, vascular, Lewy body, or other types of dementias, and having regular visits to healthcare institutions for the treatment. Based on these criteria, the Internet survey company randomly recruited members from their potential pool of participants by sending e-mails and posting notifications on their website. It was estimated that there were 1,913 persons in the pool who had a family member with a dementia diagnosis in the same household requiring regular visits to healthcare organizations for treatment.

Eligible persons who agreed to the terms and conditions of the online survey could access the self-report questionnaire. Since the Internet survey company ceased recruitment once the target number of respondents had been reached, the response rate could not be determined.

### 2.4. Measurements

All variables were measured using an online self-report questionnaire developed by the authors. The recruited participants were instructed to log in to the portal and complete the online questionnaire.

The primary outcome measure was the depressive symptoms of a person with dementia. Depressive symptoms were evaluated using an item of depression from the Neuropsychiatric Inventory – Nursing Home version (NPI-NH). The original NPI-NH comprises 12 items to rate the frequency and severity of neuropsychiatric symptoms in persons with dementia (22–25). Scores for depression range from 0 to 12, with higher scores indicating more severe symptoms. The Japanese version of the NPI-NH has good validity and reliability (26).

The secondary outcome measures included the quality of life of people with dementia. Quality of life was evaluated using the EuroQol 5 Dimension 5 Levels (EQ-5D-5L). The original EQ-5D-5L consists of five items: “mobility,” “self-care,” “usual activities,” “pain/discomfort,” and “anxiety/depression” (27, 28). The EQ-5D-5L score ranges from 0 to 1, with higher scores indicating greater quality of life. The Japanese version of the EQ-5D-5L has been validated (29, 30).

ACP initiation was the primary independent variable. In the questionnaire, family caregivers were asked whether the person with dementia had initiated ACP. ACP was defined as “thinking about one's own future, and talking to the person's family and others about what is important to the person”. The definition was created by the research team based on materials from dementia-related associations (31, 32) and suggestions from family caregivers and staff working at four dementia-related organizations in Japan. When a person with dementia did initiate any form of ACP, family caregivers were also asked to respond to the timing of initiation (e.g., diagnosis, hospital admission), types of care professionals involved in the conversation, and the topics discussed. Categories of timing of initiation were developed by the research group based on the recommendations of ACP in dementia (33). Types of professionals were defined with reference to dementia care pathways in Japan (34). Categories of topics discussed were also developed by the research group based on intervention programs to encourage ACP among community residents (35, 36). As half of the participants with ACP initiation did not involve any care professionals in the conversation, participants were divided into three groups: never initiated (*N* = 224), no professionals other than relatives involved (*N* = 67), and care professionals involved in the conversation (*N* = 88).

We measured the characteristics of persons with dementia, including age, sex, type of dementia, duration of illness from clinical diagnosis, level of cognitive impairment, activities of daily living (ADL), presence of physical complications, and delusions. Physical complications were categorized as heart disease, cancer, or circulatory disease. The level of cognitive impairment was evaluated using the Japanese version of the Cognitive Performance Scale (CPS) provided by the interRAI Assessment System (37). The is a validated measure that uses five variables to classify older adults into cognition categories ranging from intact (a score of 0) to very severely impaired (a score of 6) (37). The Japanese version of the CPS has demonstrated fair reliability and validity (38). ADL were measured using the Japanese version of the Activities of Daily Living Self-performance Hierarchy Scale (ADL-H) provided by the interRAI Assessment System (39). The ADL-H is a 10-item scale that measures basic aspects of activities related to self-care and mobility (38). Total scores range from 0 to 6, with higher scores indicating greater physical dependency. The Japanese version of the ADL-H has demonstrated good validity (40). Delusional symptoms were assessed using the NPI as they were associated with suicidal ideation (41). Duration of illness was categorized into “within 24 months”, “25–60 months”, or “61 months or longer”, based on quartiles (25, 51, 92.5) and the literature (5–7).

We also assessed characteristics of family caregivers, including age, sex, educational attainment, and relationship with the person with dementia. As the majority of family caregivers were the children (74.9%) of the person with dementia, the relationship with the person was categorized into “children” or “other relatives” in the analysis.

### 2.5. Study size

The required sample size for conducting an analysis of variance for depressive symptoms was calculated using the G^*^Power 3.1.9.7 software (42, 43). Based on recent reports on the prevalence of ACP in Belgium (11.8%) (44) and Australia (16.0%) (45), we assumed the prevalence of ACP initiation to be 16% in this study. Assuming a significance level of 0.05 and 95% power, a medium effect size (Cohen's d = 0.5), and using a two-tailed test, the desired sample size was determined to be 390.

After data collection, we discovered that out of 40.9% of the participants who had initiated ACP, 43.2% had no professionals involved in ACP; we divided the participants into three groups according to ACP initiation and professional involvement. *Post-hoc* power analysis showed a small effect size (Cohen's f = 0.2).

### 2.6. Ethical considerations

The study protocol was approved by the Ethics Committee Tohoku University Graduate School of Medicine (number 2021-5-154) and the Tokyo Metropolitan Institute of Medical Science (number 20–55). The research was conducted in accordance with the principles of the Declaration of Helsinki (version November 2013). It is in agreement with the law regarding medical-scientific research in humans.

### 2.7. Statistical analysis

The characteristics of persons with dementia and caregivers were compared among the three groups according to ACP initiation. ANOVAs were used for continuous variables with Bonferroni correction, and χ^2^ tests were used for categorical variables.

Depressive symptoms and quality of life of the person were compared among the three groups according to ACP initiation, using ANOVAs with Bonferroni correction.

Multiple linear regression analysis was conducted using the three groups according to ACP initiation as independent variables and the total score of each outcome measure as dependent variables. All the persons' and family caregivers' characteristics were included as covariates.

Statistical significance was set to α = 0.05. All statistical analyses were conducted using STATA version 17.0 (StataCorp LLC, College Station, TX, USA).

## 3. Results

### 3.1. Participant characteristics

A total of 412 family caregivers who were registered with the Internet survey company completed the survey. Of the 412 respondents, 33 were excluded because they reported that their loved ones had been admitted to long-term care facilities or hospitals by the time of the survey. Therefore, the remaining 379 family caregivers of home-dwelling individuals with dementia were included in the final sample.

At the time of enrolment, the mean age of the family caregivers was 58.2 years [standard deviation (SD) = 8.9]; 52.8% were men, 44.1% had graduated from university or graduate school, and the majority (74.9%) were children of the loved ones. Most (97.4%) of the respondents lived with their loved ones. One-fifth of the loved ones were men (19.0%; Table 1). An average of 65.3 months had passed since diagnosis (range = 2–313 months; SD = 55.8 months).

**Table 1:** Characteristics of 379 family caregivers and persons with dementia.

**Variable**	***N* (%) or mean [Standard Deviation (SD)]**
**Family caregiver**	
Age, year, range 40–83, mean (SD)	58.2 (8.9)
Sex, man, *n* (%)	200 (52.8)
**Educational attainment**, ***n*** **(%)**	
Junior high school or high school	138 (36.4)
Vocational school or college	74 (19.5)
University or graduate school	167 (44.1)
**Relationship with the person with dementia**, ***n*** **(%)**	
Child	284 (74.9)
Spouse	61 (16.1)
Spouse of child	29 (7.7)
Other relative	5 (1.3)
**Person with dementia**	
**Living situation**, ***n*** **(%)**	
Living with respondent caregiver	369 (97.4)
Living with other caregiver	3 (0.8)
Living alone	7 (1.8)
**Demographic**	
Age, year, range 41-99, mean (SD)	82.7 (8.7)
Sex, man, *n* (%)	72 (19.0)
**Duration of illness from diagnosis**, ***n*** **(%)**	
Within 24 months	92 (24.3)
25–60 months	120 (31.7)
61 months or longer	167 (44.1)
**Type of dementia**, ***n*** **(%)**	
Alzheimer's disease	254 (67.0)
Vascular	55 (14.5)
Lewy body	51 (13.5)
Frontotemporal	9 (2.4)
Mixed	13 (3.4)
Other, including unspecified	24 (6.3)
**Functioning**	
ADL dependence, range 0–6, mean (SD)	2.9 (2.0)
Cognitive impairment, range 0–6, mean (SD)	3.1 (1.2)
Delusional symptoms, range 0–12, mean (SD)	1.5 (2.9)
**Physical complication**, ***n*** **(%)**	
Cardiovascular disease	76 (20.1)
Neurological disease other than Alzheimer's disease	54 (14.2)
Respiratory disease	25 (6.6)
Malignant neoplasm	19 (5.0)
Kidney disease	16 (4.2)

Activities of daily living (ADL) were evaluated using the Japanese version of the Activities of Daily Living Self-Performance Hierarchy Scale. Cognitive impairment was evaluated using the Japanese version of the Cognitive Performance Scale. Delusional symptoms were evaluated using the Japanese version of the Neuropsychiatric Inventory (NPI).

### 3.2. Initiation of ACP

One hundred and fifty-five (40.9%) of the respondents reported that their loved ones had initiated ACP. Of these, approximately half (49.7%) reported that the initiation was triggered by a dementia diagnosis. One-fifth (20.0%) reported that the initiation was triggered by new accreditation for a long-term care insurance benefit. Other types of triggers included increased difficulty managing own property or daily life (8.4%) and admittance to an acute hospital for treatment of a physical illness (4.5%).

Two-fifths (43.2%) reported that no one other than relatives were involved in the ACP conversations. Regarding the cases in which health-care professionals were involved in such conversations, the most commonly reported type of professional was care managers of in-home care services (34.2%). Other types of professionals included staff of a day-care center (27.7%), care manager of a residential care service (25.2%), and Community General Support Center (17.4%) which provides comprehensive support for older community residents. The doctor who provided the dementia diagnosis was present in 16.8% of the cases. Initial-phase intensive support team for dementia that, conducts home visits and assessments, and provides information and advice to persons with early signs of dementia, was present in only 5.2% of the cases.

The most frequently discussed ACP-related topic was the point at which the loved ones would need to enter residential care (67.7%). Other topics included important roles in the community and values (41.9%), the loved one's habits and preferences (38.7%), social activities the loved one would like to continue (29.0%), social relationships the loved one would like to maintain (25.2%), tube feeding (25.2%), and application of cardiopulmonary resuscitation or transfer to an emergency department when breathing or heart stops (21.9%).

The 88 persons whose care professionals were involved in the conversation had significantly older caregivers, male caregivers, caregivers with higher educational attainment, more severe ADL dependence, more severe delusional symptoms, and a higher prevalence of respiratory disease or malignant neoplasm (Supplementary Table 1).

### 3.3. Depressive symptoms of persons with dementia

The mean score of depressive symptoms measured by NPI was 1.34 (SD = 2.46). A total of 143 persons (37.7%) presented with depressive symptoms. The 88 persons with professional involvement showed significantly more severe depressive symptoms compared to 224 persons without ACP initiation. There was no significant difference in depressive symptoms between the 67 persons with no professional involvement and 224 persons without initiation (Table 2).

**Table 2:** Comparison of outcome measures according to the initiation of advance care planning (ACP).

	**ACP initiation**				
**Mean (standard deviation)**	**Never initiated (*****N** =* **224)**	**No professionals involved (*****N** =* **67)**	**Professionals involved (*****N** =* **88)**	**F (2)**	***P*** **-value**
Depressive symptoms	0.99 (2.02)^a^	1.33 (2.40)	2.24 (3.21)^a^	8.52	< 0.001
Quality of life	0.64 (0.21)^a^	0.64 (0.25)	0.56 (0.22)^a^	4.63	0.010

^a^Significant difference with P < 0.017, Bonferroni correction. Depressive symptoms were evaluated using the Japanese version of the Neuropsychiatric Inventory (NPI) (range 0–12). Quality of life was evaluated using the Japanese version of the EuroQol 5 dimensions 5-level (EQ-5D-5L) (range 0–1).

Multiple linear regression analysis showed a significant association with more severe depressive symptoms in persons with professional involvement (Table 3). There were also significant associations with more severe depressive symptoms in more severe delusional symptoms and presence of neurological diseases other than Alzheimer's disease (Supplementary Table 2).

**Table 3:** Multiple linear regression analyses of outcome measures.

	**Depressive symptoms**		**Quality of life**	
	**Coefficient**	**95%CI**	**Coefficient**	**95%CI**
ACP initiation, reference = never initiated				
Professionals involved	0.69	0.11, 1.26	0.003	−0.04, 0.04
No professionals involved	0.001	−0.60, 0.60	−0.01	−0.05, 0.03

CI, confidence interval. Depressive symptoms were evaluated using the Japanese version of the Neuropsychiatric Inventory (NPI) (range 0–12). Quality of life was evaluated using the Japanese version of the EuroQol 5 dimensions 5-level (EQ-5D-5L) (range 0–1). The model included following covariates: family caregiver's age, sex, educational attainment; the person with dementia's age, sex, duration of illness from diagnosis, type of dementia, ADL dependence, cognitive impairment, delusional symptoms, and physical complication.

### 3.4. Quality of life of persons with dementia

The mean score of quality of life measured by the EQ-5D-5L was 0.62 (SD = 0.22). The 88 persons with professional involvement showed significantly lower quality of life compared to 224 persons without ACP initiation. There was no significant difference in quality of life between the 67 persons with no professional involvement and 224 persons without initiation (Table 2).

The multiple linear regression analysis did not show significant association between quality of life and ACP initiation (Table 3). Quality of life was significantly greater for younger persons with dementia, women, those who had a shorter duration of illness from diagnosis, less dependence on ADL, less cognitive impairment, less severe delusional symptoms, and absence of neurological disease, malignant neoplasm, or kidney disease (Supplementary Table 2).

## 4. Discussion

Contrary to our hypothesis, ACP initiation was associated with more severe depressive symptoms in home-dwelling persons with dementia. ACP initiation was not significantly associated with the quality of life of persons with dementia. Initiation was triggered by dementia diagnosis or accreditation of long-term care insurance benefits. Among those with initiation, 43% did not have any care professionals involved in the conversation process. Furthermore, professional involvement was significantly associated with worse depressive symptoms in persons with dementia.

There is little evidence regarding person-centered outcomes of ACP in terms of depressive symptoms or quality of life, rather than end-of-life measures (14, 46). One randomized-controlled study reported greater improvement in depressive symptoms and quality of life among 10 persons with dementia who received the intervention (35). The association between depressive symptoms and ACP initiation in this study was inconsistent with the previous study (35). There are long-term concerns among professionals regarding ACP causing stress and anxiety in people with dementia and family caregivers (14). This is because the conversation process requires imagining a situation in which the person will lose decisional capacity. However, trusting and open relationships would help overcome such difficult emotions (47). Our findings showed that the participants had a mean duration of 65 months following the clinical diagnosis of dementia, and 39% had depressive symptoms. Although the risk of suicide is most likely after initial diagnosis and decreases over time (5–7), depression and anxiety are highly prevalent across dementia stages (48). Many persons in our study experienced depressive symptoms even some years after receiving the dementia diagnosis. Thus, the ACP initiation and involvement of professionals could reflect their coping strategies in response to dementia-related anxiety. Anxiety and depression were associated with greater ACP engagement among older adults (49). Our results suggest that care professionals may intervene only when the person has worsened symptoms. Further examination is needed to verify whether optimal, timely, and proactive ACP approaches in newly diagnosed persons can prevent dementia-related anxiety and depressive symptoms.

Care professionals were involved in conversations with 57% of the 155 persons with dementia who had initiated ACP. The most frequent types of care professionals involved in ACP included care managers of in-home care services (34%), staff of day-care centers (28%), and care managers of residential care services (25%). Only 17% of the participants had conversations involving doctors who provided the dementia diagnosis. In Japan, where there is no registration system for general practitioners, initiation of ACP is promoted in the Initial-phase Intensive Support Teams, which are expected to provide post-diagnostic support to people with dementia and family caregivers (34). However, in our study, there were only 5% of participants whose ACP involved Initial-phase Intensive Support Teams. This may reflect the fact that most people with dementia do not access initial-phase intensive support teams due to less availability compared to general health and social care services.

Our findings also showed that 40.9% of the participants had initiated ACP. The involvement of the person with dementia appeared to be greater than that reported by family physicians among nursing home residents in Belgium (11.8%) (44). Since, 2018 the Ministry of Health, Labor, and Welfare in Japan has announced November 30 as the national ACP (Jinsei-Kaigi) day. This national campaign may have increased awareness of ACP among community-dwelling older adults (50). Uncertainty and instability in healthcare due to the COVID-19 pandemic could also add to the awareness of persons with dementia and family caregivers about planning for the future. Nonetheless, the topics discussed were focused on institutionalization rather than living well with dementia, such as important roles in the community and values of the person. In Australia, more than a half of individuals with dementia knew about ACP, whereas only one-quarter had written down their values and preferences for future care (51). Most persons with dementia in Canada preferred focusing on the present rather than planning for the future (52). Therefore, raising awareness strategies may be imperative to encourage people with dementia to express their values and future hope during ACP conversations. There is currently limited evidence on effective ways of engaging persons with dementia in ACP (53). The COVID-19 guidance on ACP has largely focused on a plan recording an individual's treatment process rather than enabling conversations that constitute the planning process (54). Further strategies are warranted to implement ACP in a more ethical, coordinated and person-centered practice during the COVID-19 pandemic (55).

### 4.1. Strengths and limitations

The strengths of this study lie in the inclusion of home-dwelling persons and person-centered outcome measures of ACP rather than measures specific to end-of-life care. However, our study has some limitations. A cross-sectional design could not determine the causality between ACP initiation and depressive symptoms. Our data collection was based on the responses of family caregivers. This may have led to a bias regarding outcome measures for persons with dementia and the initiation of ACP. Family caregivers tended to rate worse quality of life for the person with dementia than their self-reported evaluation (56). Family caregivers also had a low to moderate agreement with persons with dementia on care preferences (57). This might have affected their perception of the ACP initiation and types of professionals involved in conversation. Although sex and mean age of our participants were similar with those reported in previous studies using online surveys in Japan (58, 59), our participants included more sons than female partners and daughters of people with dementia, which were more often reported in clinical settings (60) and national questionnaire surveys (61). This study's definition of ACP was developed based on suggestions from Japanese dementia-related associations with a focus on conversation. This definition may not be applicable to ACP in other countries that typically involve documentation of preferences and decisions.

## 5. Conclusion

Persons with dementia exhibited more severe depressive symptoms when they had care professionals involved in ACP. This study is an important step toward improving post-diagnostic support for community-dwelling persons having dementia and their family caregivers, requiring the implementation of a more person-centered approach to ACP. Educational and clinical strategies should be examined to encourage care professionals to engage in proactive and effective ACP for people with dementia in the early stages of the disease course.

## Data availability statement

The raw data supporting the conclusions of this article will be made available by the authors, without undue reservation.

## Ethics statement

The studies involving human participants were reviewed and approved by the Ethics Committee Tohoku University Graduate School of Medicine, Tokyo Metropolitan Institute of Medical Science. The patients/participants provided their informed consent to participate in this study.

## Author contributions

MN, TN, YM, and SY devised the project, conceptualized the ideas, participated in the study design, and evaluation of the questionnaire. MS, HY, and AN were involved in the literature review. MN and TN assisted in data collection and data entry. MN and SY performed data analysis and drafting of the manuscript. TN, YM, MS, HY, SY, and AN contributed substantially to manuscript revision. All authors have read, agreed to the published version of the manuscript, contributed to the article, and approved the submitted version.
