# Supplementary material for: Association between advance care planning and depressive symptoms among community-dwelling people with dementia: An observational cross-sectional study during the COVID-19 pandemic in Japan
Source: Front Public Health. 2023 Mar 30;11:915387. doi: 10.3389/fpubh.2023.915387 (PMC10098156; doi:10.3389/fpubh.2023.915387)
Supplement: Supplementary file 1 [file Table_1.DOCX]

Supplementary Material

# Supplementary Tables

**Supplementary Table 1** Comparison of characteristics according to the advance care planning (ACP) initiation

|  | ACP initiation |  |  |  |  |
| --- | --- | --- | --- | --- | --- |
| Variable | Never initiated (N = 224) | No professionals involved (N = 67) | Professionals involved (N = 88) | Test statistics | P-value |
| Family caregiver |  |  |  |  |  |
| Age, year, range 40–83, mean (SD) | 58.6 (8.8)^a^ | 55.6 (9.3)^a,b^ | 59.1 (8.8)^b^ | F (2) = 3.59 | .028 |
| Sex, man, n (%) | 106 (47.3) | 34 (50.7) | 60 (68.2) | χ^2^ (1) = 11.16 | .004 |
| Educational attainment, n (%) |  |  |  | z = 8.84 | .012 |
| Junior high school or high school | 90 (40.2) | 24 (35.8) | 24 (27.3) |  |  |
| Vocational school or college | 49 (21.9) | 12 (17.9) | 13 (14.8) |  |  |
| University or graduate school | 85 (37.9) | 31 (46.3) | 51 (58.0) |  |  |
| Child of the person with dementia, n (%) | 165 (73.7) | 53 (79.1) | 66 (75.0) | χ^2^ (1) = 0.81 | .666 |
| Person with dementia |  |  |  |  |  |
| Age, year, range 41-99, mean (SD) | 82.8 (8.1) | 82.0 (8.8) | 82.7 (10.1) | F (2) = 0.23 | .793 |
| Sex, man, n (%) | 44 (19.6) | 12 (17.9) | 16 (18.2) | χ^2^ (1) = 0.15 | .928 |
| Duration of illness from diagnosis, n (%) |  |  |  | z = 0.24 | .885 |
| Within 24 months | 57 (25.4) | 14 (20.9) | 21 (23.9) |  |  |
| 25-60 months | 67 (29.9) | 27 (40.3) | 26 (29.5) |  |  |
| 61 months or longer | 100 (44.6) | 26 (38.8) | 41 (46.6) |  |  |
| Alzheimer’s disease, n (%) | 141 (62.9) | 48 (71.6) | 65 (73.9) | χ^2^ (1) = 4.19 | .123 |
| ADL dependence, range 0-6, mean (SD) | 2.8 (2.0)^a^ | 2.5 (2.1)^b^ | 3.5 (2.0)^a,b^ | F (2) = 6.31 | .002 |
| Cognitive impairment, range 0-6, mean (SD) | 3.1 (1.3) | 3.1 (1.3) | 3.3 (1.2) | F (2) = 1.45 | .237 |
| Delusional symptoms, range 0-12, mean (SD) | 1.1 (2.5)^a^ | 1.9 (3.1) | 2.3 (3.4)^a^ | F (2) = 5.85 | .003 |
| Physical complication, n (%) |  |  |  |  |  |
| Cardiovascular disease | 37 (16.5) | 14 (20.9) | 25 (28.4) | χ^2^ (1) = 5.61 | .061 |
| Neurological disease other than Alzheimer’s disease | 32 (14.3) | 8 (11.9) | 14 (15.9) | χ^2^ (1) = 0.49 | .782 |
| Respiratory disease | 10 (4.5) | 3 (4.5) | 12 (13.6) | χ^2^ (1) = 9.22 | .010 |
| Malignant neoplasm | 4 (1.8) | 1 (1.5) | 14 (15.9) | χ^2^ (1) = 28.58 | <.001 |
| Kidney disease | 7 (3.1) | 2 (3.0) | 7 (8.0) | χ^2^ (1) = 3.95 | .139 |

^a,b^ Significant difference with P < .05, Bonferroni correction.

Activities of daily living were evaluated using the Japanese version of the Activities of Daily Living Self-Performance Hierarchy Scale.

Cognitive impairment was evaluated using the Japanese version of the Cognitive Performance Scale.

Delusional symptoms were evaluated using the Japanese version of the Neuropsychiatric Inventory (NPI).

**Supplementary Table 2** All covariates in multiple linear regression analyses of outcome measures

|  | Depression |  | Quality of life |  |
| --- | --- | --- | --- | --- |
| Variable | Coefficient | 95%CI | Coefficient | 95%CI |
| Family caregiver |  |  |  |  |
| Age, year | -0.02 | -0.05, 0.01 | 0.001 | -0.001, 0.003 |
| Sex, man | -0.13 | -0.62, 0.36 | -0.03 | -0.06, 0.003 |
| Educational attainment, reference = junior high school or high school |  |  |  |  |
| Vocational school or college | -0.17 | -0.79, 0.44 | 0.001 | -0.04, 0.04 |
| University or graduate school | -0.13 | -0.64, 0.38 | -0.01 | -0.05, 0.02 |
| Child of the person with dementia | -0.28 | -0.92, 0.37 | 0.04 | -0.01, 0.08 |
| Person with dementia |  |  |  |  |
| Age, year | -0.01 | -0.04, 0.02 | -0.002 | -0.005, -0.0004 |
| Sex, man | -0.32 | -0.93, 0.28 | -0.04 | -0.08, 0.004 |
| Duration of illness from diagnosis, reference = longer than 5 years |  |  |  |  |
| Within 24 months | -0.04 | -0.63, 0.55 | 0.05 | 0.01, 0.09 |
| 25-60 months | 0.06 | -0.47, 0.60 | 0.04 | 0.001, 0.07 |
| Alzheimer’s disease | 0.35 | -0.12, 0.82 | 0.02 | -0.02, 0.05 |
| ADL independence, range 0-6 | -0.03 | -0.16, 0.10 | -0.05 | -0.06, -0.04 |
| Cognitive impairment, range 0-6 | 0.13 | -0.08, 0.34 | -0.04 | -0.06, -0.03 |
| Delusional symptoms, range 0-12 | 0.32 | 0.24, 0.40 | -0.01 | -0.02, -0.004 |
| Physical complication |  |  |  |  |
| Cardiovascular disease | 0.40 | -0.17, 0.97 | 0.01 | -0.03, 0.04 |
| Neurological disease other than Alzheimer’s disease | 0.80 | 0.15, 1.46 | -0.08 | -0.13, -0.04 |
| Respiratory disease | -0.15 | -1.10, 0.81 | -0.01 | -0.07, 0.06 |
| Malignant neoplasm | 0.78 | -0.33, 1.89 | -0.08 | -0.16, -0.01 |
| Kidney disease | 0.85 | -0.35, 2.05 | -0.09 | -0.17, -0.01 |

Activities of daily living (ADL) were evaluated using the Japanese version of the Activities of Daily Living Self-Performance Hierarchy Scale.

Cognitive impairment was evaluated using the Japanese version of the Cognitive Performance Scale.

Delusional symptoms were evaluated using the Japanese version of the Neuropsychiatric Inventory (NPI).
